# Wheat Yield, N Use Efficiency, Soil Properties, and Soil Bacterial Community as Affected by Long-Term Straw Incorporation and Manure Under Wheat–Summer Maize Cropping System in Southern Shanxi Province, China

**DOI:** 10.3390/plants14121795

**Published:** 2025-06-11

**Authors:** Mengni Chen, Zhiguo Yang, Na Yang, Hui Wang, Yongshan Li, Ke Wang, Jian Wang, Qiaolan Fan, Jiancheng Zhang, Jiawei Yuan, Peng Dong, Lu Wang

**Affiliations:** Key Laboratory of Sustainable Dryland Agriculture of Shanxi Province, Cotton Research Institute, Shanxi Agricultural University, Yuncheng 044000, China

**Keywords:** straw incorporation, manure, yield, N use efficiency, soil fertility, bacterial community

## Abstract

Straw incorporation and manure are recognized as a sustainable farming practice to enhance soil fertility and improve crop yields. However, the effects of straw incorporation in combination with manure on productivity, soil nutrient status, N use efficiency (NUE), and the bacterial community are not well understood in wheat–summer maize rotation systems in the southern Shanxi Province. The five treatments were (1) CK, no fertilization; (2) NP, inorganic N and P fertilizers; (3) NPM, mineral N and P fertilizers plus chicken manure; (4) SNP, mineral N and P fertilizers plus maize straw; and (5) SNPM, mineral N and P fertilizers plus maize straw and chicken manure. The results showed that NP, NPM, SNP, and SNPM significantly increased wheat yields by 56.19%, 76.89%, 111.08%, and 114.30%, compared with CK, respectively. Nitrogen agronomic efficiency (AE_N_), partial factor productivity (PEP_N_), apparent recovery efficiency (Apparent RE_N_), and accumulated recovery efficiency (Accumulated RE_N_) increased by 103.36%, 37.19%, 76.39%, and 30.90% in the SNPM treatment, compared with NP. Straw incorporation and manure significantly improved soil fertility. *Proteobacteria*, *Acidobacteriota*, *Actinobacteriota*, *Chloroflex*, *Bacteroidota*, *Planctomycetota*, *Gemmatimonadota*, *Armatimonadota*, *Firmicutes*, *Methylomirabilota*, and *Myxococcota* were the predominant bacterial phyla. Compared with NP, straw incorporation and manure (NPM, SNP, and SNPM) decreased diversities (richness index, Chao1 index, and Shannon index). Principal coordinates (PCoA) and cluster analyses demonstrated that manure treatments (NPM and SNPM) significantly optimized bacterial community structure. Pearson’s correlation analysis demonstrated that organic matter, total phosphorus, available nitrogen, available phosphorus, and available potassium had significant positive correlations with *Halanaerobiaeota* but significant negative positive correlations with *Chloroflexi, Entotheonellaeota,* and *Myxococcota*. Wheat yields, AE_N_, PEP_N_, Apparent RE_N_, and Accumulated RE_N_ were primarily and significantly negatively associated with *Cyanobacteria.* Straw incorporation in combination with manure significantly optimized bacterial community structure, wheat yields, and N use efficiency through improving soil fertility. Collectively, straw incorporation in combination with manure is a promising practice for sustainable development.

## 1. Introduction

In order to meet increasing food demand for a growing population, excessive chemical fertilizer application to maximize yields has caused serious soil degradation and environmental pollution [1,2], and reduced fertilizer use efficiency [3]. The adoption of sustainable agricultural practices significantly contributes to soil quality improvement and ensures global food security on a global scale [4]. Straw incorporation and manure have been recognized as a sustainable farming practice and an efficient strategy to enhance soil fertility, improve soil structure, and increasing crop yields [5,6,7,8]. During the agricultural production process in China, 2.4 × 10^9^ tons (t) of animal manure are generated each year [9], and 977 million tons of crop straw resources were produced in 2023 [10]. Straw incorporation can effectively improve soil organic carbon [11] and enhance soil structure, regulate soil enzyme activities, and increase soil nutrient availability and crop yields [12,13,14,15,16,17,18,19]. Furthermore, manure can significantly improve soil fertility, and increase crop yields [20,21,22,23,24,25,26,27,28,29,30]. Additionally, straw incorporation and manure tackle environmental problems associated with straw burning and nitrogen-rich waste management [31,32,33,34,35]. Therefore, straw incorporation and manure application are better practices for soil sustainable development.

Soil bacteria plays important roles as the major driving force in soil organic matter and nutrient cycling [36]. Straw incorporation-affected soil bacterial communities have been investigated worldwide. Zhao et al. [37] showed straw incorporation increased the Gram-negative (G−) bacterial abundance, but had no obvious effects on Gram-positive (G+) bacteria in a 30-year maize–wheat cropping system. Another study revealed that bacterial abundance, composition, and diversity were significantly impacted by straw incorporation in a rice–wheat cropping system [38]. Wu et al. [39] revealed that straw incorporation increased bacterial diversity and soil multifunctionality. Our previous research found that straw incorporation promoted the abundance of PLFA-labeled microorganisms, and slightly altered the composition of microbial communities in wheat–summer maize systems on the Loess Plateau [40]. However, Yu et al. [41] indicated that straw incorporation had no influence on bacterial abundance, richness, or diversity. Hence, the effect of straw incorporation on the soil bacterial community remains controversial and worthy of investigation.

Manure has been shown to dominate in shaping bacterial community distributions [42], enhance root exudates to promote soil bacterial communities, and improve plant growth [43]. Wu et al. [44] revealed that manure application altered the bacterial community’s composition and structure. Zhang et al. [45] found that manure increased the Gram bacterial (G+ and G−) abundance. Tian et al. [46] showed that manure significantly increased the abundance of total PLFAs, bacteria, and actinomycetes. Wei et al. [47] found that manure significantly reduced the ratio of fungal to bacterial biomass and changed the bacterial community. Zhang et al. [48] indicated that cattle manure promoted bacterial consumption of labile C, especially *Gemmatimonadetes* and *Acidobacteria*. Hu et al. [49] found that organic manure application significantly increased the abundance of species associated with C-N-P-S and enhanced soil multifunctionality. Manure increased functional gene abundances, which is involved in C fixation, C degradation, and N fixation nitrification as well as P transporters [50,51]. Organic amendments significantly increased microbial diversity components and shifted the microbial community’s structure [52]. Cui et al. [53] found that organic amendments increased the bacterial Shannon and Chao1 diversity indices and copiotrophic strategies such as *Proteobacteria* and *Bacteroidetes* phylum, even producing a more abundant and uniform bacterial community [54,55] and increasing bacterial diversity [56]. However, Tian et al. [57] reported that organic compost significantly decreased diversity. The effects of soil microbe to organic amendments were significantly varied by many factors such as soil texture, manure type, crops, and climate [53].

Some reports suggest that manure in combination with straw incorporation could improve soil fertility and crop yield [30,58]. However, the effects of straw incorporation in combination with manure on crop yield, soil nutrient status, N use efficiency, and the bacterial community are still unclear in winter wheat–summer corn rotation systems in the southern Shanxi Province on the Loess Plateau. Therefore, we hypothesized that straw incorporation in combination with manure can improve soil nutrient status and crop yield, increase N use efficiency, and alter the soil bacterial community. The objective of this research is to evaluate the changes in wheat yield, N use efficiency, soil fertility, and the soil bacterial community in the region under application of manure and straw incorporation.

## 2. Results

### 2.1. Wheat Grain Yield and N Use Efficiency

Analysis of variance (ANOVA) showed that straw incorporation and manure treatments significantly affected grain yield and nitrogen use efficiency (NUE) (Table 1). Comparison with the control (CK), the SNPM treatment significantly increased grain yields by 114.30%, followed by SNP with 111.08%, NPM with 76.89%, and NP with 56.19% (*p* < 0.05). Both straw incorporation and manure treatments significantly improved NUE indicators (AE_N_, PEP_N_, Apparent RE_N_, and Accumulated RE_N_) (*p* < 0.05). Compared with the NP treatment, AE_N_, PEP_N_, apparent RE_N_, and accumulated RE_N_ improved by 103.36%, 37.19%, 76.39%, and 30.91% in the SNPM treatment, respectively. The SNP and SNPM treatments did not show significant differences in these NUE indicators, whereas other treatments revealed significant differences in the NUE indicators (*p* < 0.05).

### 2.2. Soil Chemical Properties

The effects of different fertilization regimes on the soil chemical properties are summarized in Table 2. Fertilization significantly decreased the soil pH compared with CK (*p* < 0.05). The soil pH ranged from 7.86 in the SNPM treatment to 8.07 in CK treatment. Soil organic matter (OM) significantly increased by 23.35–81.72% in manure and straw-treated soils (NPM, SNPM, and SNP), compared with CK (*p* < 0.05). Straw incorporation and manure increased the soil total nitrogen (TN), especially in the SNP and SNPM treatments, but there were no obvious differences among the treatments (*p* > 0.05). Manure and straw incorporation significantly increased soil available nitrogen (AN) by 51.98–146.06%, in the NPM, SNP, and SNPM treatments, compared with the CK treatment, respectively. Importantly, the SNPM treatment significantly increased by 4.8, 13.4, and 2.2 times more than the CK treatment in total phosphorus (TP) content, available phosphorus (AP) and available potassium (AK), respectively.

### 2.3. OTU Richness and Alpha-Diversity

**Figure 1:**
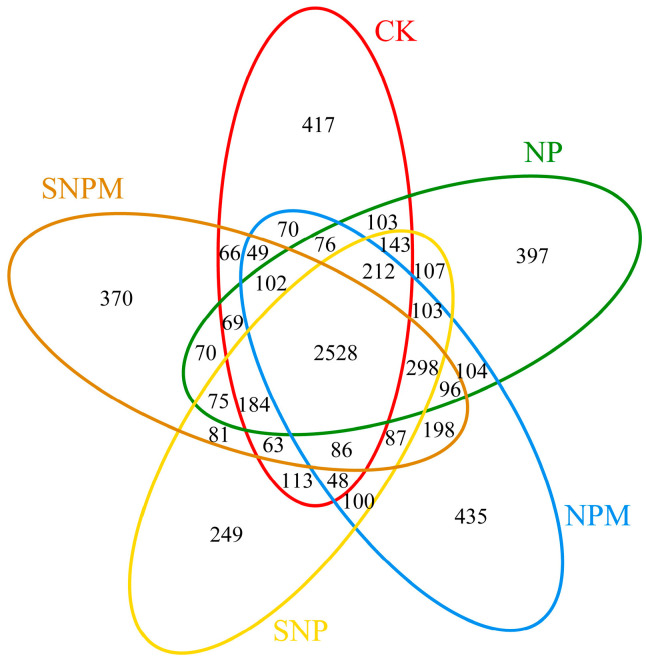
The bacterial Venn diagram in the different treatments.

MiSeq sequencing showed that a total of 7099 OTUs were obtained from the 15 samples and 2528 OTUs were shared in all soil samples (Figure 1). NP, NPM, and SNPM had significantly higher OTUs compared with CK, while there was no significant difference between the CK and SNP treatments (*p* > 0.05) (Figure 1).

Based on the OTUs, there were significant differences (*p* < 0.05) in the alpha-diversity of different straw incorporation and manure treatments (Table 3). The NP and NPM treatments had higher values of community richness index and Chao1, whereas SNP and SNPM had lower alpha-diversities, and NP, NPM, SNP, and SNPM were higher than CK. The Shannon index was remarkably higher in the NP treatment than CK, but there were no obvious differences between NP, NPM, SNP, and SNPM. No significant differences were found in the ACE index under all treatments.

### 2.4. Composition and Structure of Bacteria

**Figure 2:**
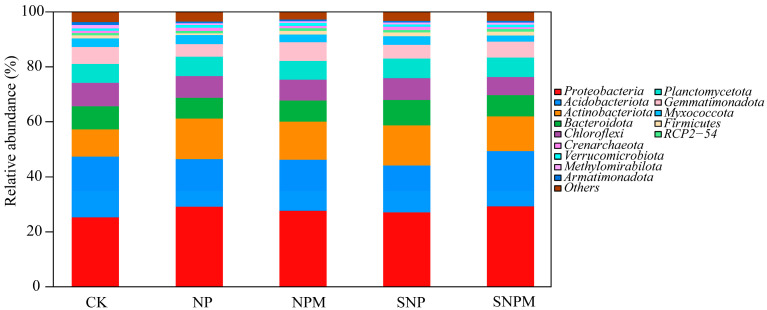
Soil bacterial relative abundance of different treatments at phylum level.

The bacterial OTUs were detected and sorted into 40 phyla, 100 classes, 213 orders, 272 families, 488 genera, and 591 species. The fertilization regimes altered the composition of bacterial communities at the phylum and genus levels. Proteobacteria, Acidobacteriota, Actinobacteriota, Chloroflex, Bacteroidota, Planctomycetota, Gemmatimonadota, Armatimonadota, Firmicutes, Methylomirabilota, and Myxococcota were the top 11 most abundant bacterial phyla in all samples (Figure 2). Together, these phyla accounted for 92.76–93.66% of the total sequences. Compared with the CK treatment, the relative abundance of Proteobacteria and Actinobacteriota significantly increased by 7.08–15.91% and 27.25–48.69% in the NP, NPM, SNP, and SNPM treatments, respectively, while Acidobacteriota, Chloroflexi, Armatimonadota, and Methylomirabilota significantly decreased by 9.02–22.49%, 7.25–23.03%, 43.05–56.19%, and 35.71–45.95% in the NP, NPM, SNP, and SNPM treatments (*p* < 0.05), respectively. The relative abundance of Bacteroidota decreased by 10.39%, 8.47%, and 7,65% in the NP, NPM, and SNPM treatments, respectively, while it increased by 10.68% in the SNP treatment (*p* < 0.05). The relative abundance of Firmicutes obviously decreased by 42.60% in the NP treatment, while it significantly increased by 20.75%, 22.74%, and 26.05% in the NPM, SNP, and SNPM treatments (*p* < 0.05), respectively. The relative abundance of Gemmatimonadota significantly decreased by 25.05% and 18.90% in the NP and SNP treatments, whereas it increased by 11.22% in the NPM treatment (*p* < 0.05).

**Figure 3:**
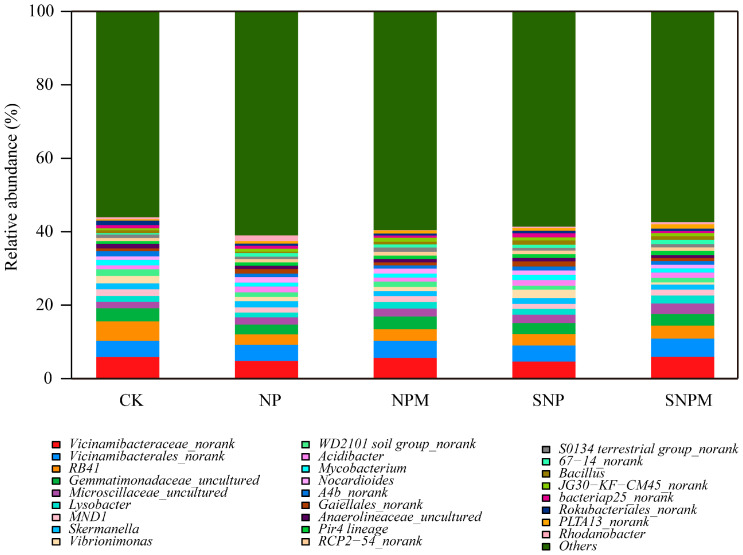
Soil bacterial relative abundance of treatments at genus level.

At the genus level, over 846 genera were identified (Figure 3); in particular, the relative abundances of the top 15 genera were greater than 1%. Vicinamibacteraceae_norank (4.65–5.96%), RB41 (2.86–5.33%), Vicinamibacterales_norank (4.32–4.94%), Gemmatimonadaceae_uncultured (2.67–3.57%), and Vibrionimonas (0.65–2.25%) were the dominant genera, accounting for 15.86–21.23%. Compared to the CK treatment, the relative abundance of Vicinamibacteraceae_norank obviously decreased by 18.20%, 5.00%, and 21.54% in the NP, NPM, and SNP treatments (*p* < 0.05), respectively, whereas it increased by 0.46% in the SNPM treatment (*p* > 0.05). The relative abundance of RB41 significantly decreased by 46.24%, 40.11%, 41.04%, and 33.94% in the NP, NPM, SNP, and SNPM treatments (*p* < 0.05), respectively. The relative abundance of Microscillaceae_uncultured significantly increased by 18.18%, 27.86%, 35.63%, and 70.67% in the NP, NPM, SNP, and SNPM treatments (*p* < 0.05), respectively.

**Figure 4:**
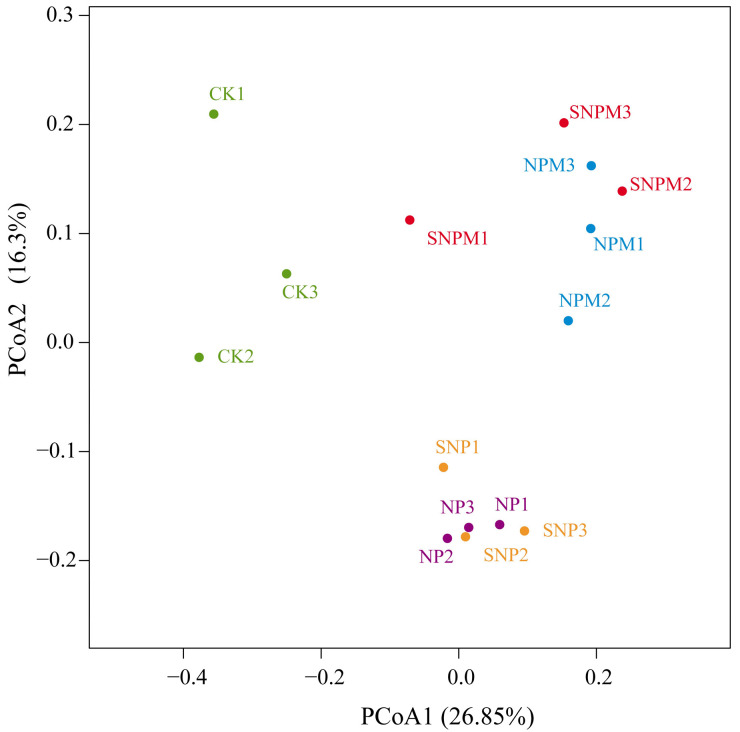
Bacterial community principal coordinates analysis (PCoA) based on Bray–Curtis distances at OTU level (97% sequence similarity).

Principal coordinates analysis (PCoA) showed that the first and second axes explained 26.85% and 16.3% of the total variation in all bacterial community composition, respectively (Figure 4). The bacterial community structure was significantly affected by manure and straw incorporation. PCoA plots showed that bacterial community composition was distinctly separated into three groups and that the manure treatments (NPM and SNPM) were obviously separate from the fertilizer treatments (NP, SNP) and CK.

**Figure 5:**
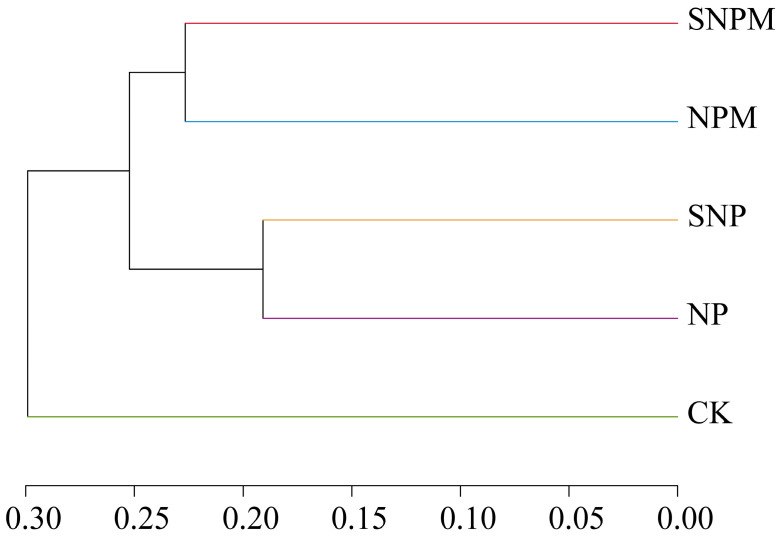
Hierarchical cluster analysis of bacterial community at the OTU level.

Cluster analysis showed that the treatments were clustered into three catalogues: the first was CK, the second contained NP and SNP, and the third included NPM and SNPM. Therefore, applied manure was the most important factor affecting bacterial community (Figure 5).

**Figure 6:**
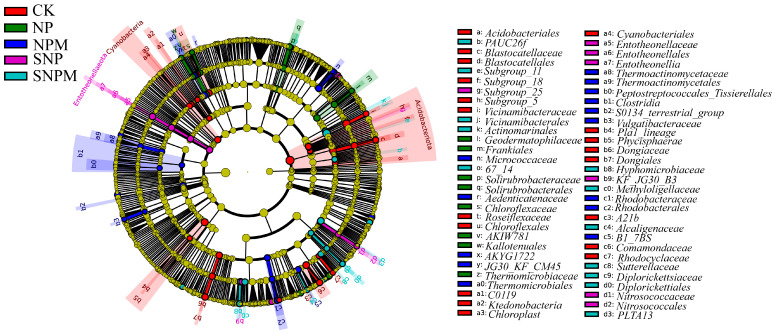
Soil bacteria community LEfSe analysis in different treatments. Color nodes indicate the taxa under different treatments. The diameter of each node shows the relative abundance of each taxon.

LEfSe analysis showed that a total of 95 significant biomarkers were detected in the bacterial microbial community among the five fertilization regimes (Figure 6). There were 27 significant biomarkers in CK and 15 significant biomarkers in NP. There were 21 significant biomarkers in NPM, 21 significant biomarkers in SNP, and 20 significant biomarkers in SNPM. Proteobacteria and Acidobacteriota were significantly enriched in CK. Actinobacteriota and Chloroflexi were significantly enriched in NP. Entotheonellaeota and Proteobacteria were significantly enriched in SNP. Proteobacteria, Firmicutes, and Chloroflexi were significantly enriched in NPM. Proteobacteria and Acidobacteriota were significantly enriched in SNPM. These results indicated that SNPM and NPM changed the soil bacterial microbial species composition.

### 2.5. Bacterial Community Relationships with Soil Properties, Wheat Yield, and NUE

**Figure 7:**
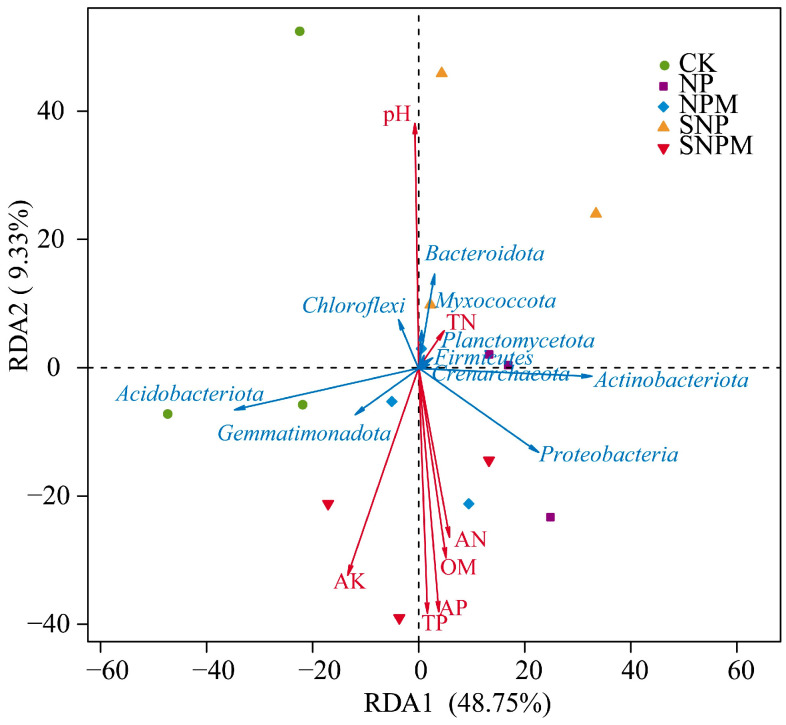
Redundancy analysis (RDA) between soil bacterial community and soil properties.

The top 10 most abundant bacterial phyla were selected using redundancy analysis (RDA) to evaluate the relationship between the soil properties and the bacterial community structure. RDA indicated that the first and second ordination axes (RDA1) explained 48.75% and 9.33% of the variation, meaning that they explained a total of 58.08% of the variation together (Figure 7). The pH, TP, AP, AK, OM, and AN appeared to be the most important factors influencing the bacterial community structure.

**Figure 8:**
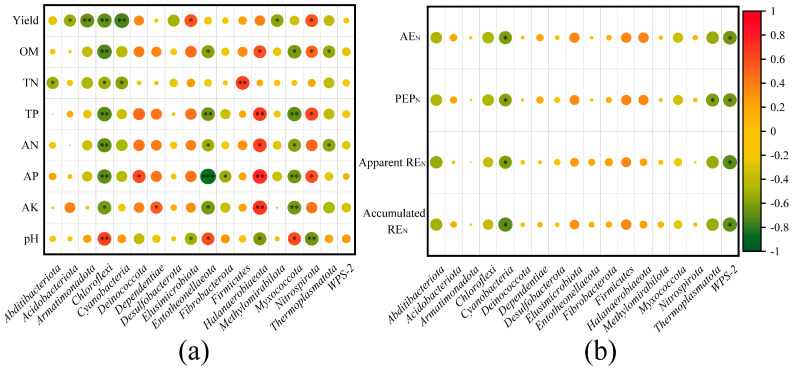
Correlation analysis of the bacterial community relationships with soil properties and yield (**a**) and NUE (**b**). *, **, and *** denote significant differences at *p* < 0.05, *p* < 0.01, and *p* < 0.001, respectively.

Pearson’s correlation analysis was carried out for the bacterial community with soil properties, yield, and NUE at the phylum (Figure 8). TN showed a significant positive correlation with Firmicutes and was negatively correlated with Abditibacteriota, Armatimonadota, Chloroflexi, and Cyanobacteria. Abditibacteriota exhibited a significant negative correlation with Apprent REN. Chloroflexi showed a significant positive relationship with pH and was negatively correlated with OM, TN, TP, AN, AP, AK, and wheat yield. Cyanobacteria displayed significant negative correlations with NUE indicators and wheat yield. Nitrospirota showed significant positive correlation with OM, TP, AP, and wheat yield, but was negatively correlated with pH. Acidobacteriota had significantly negative correlation with yield. Halanaerobiaeota showed positive correlation with OM, TP, AN, AP, and AK and was negatively correlated with pH. Myxococcota and Entotheonellaeota were positively associated with pH and negatively associated with OM, TP, AN, AP, and AK. Thermoplasmatota displayed negative correlation with OM, AN, and PEP_N_. WPS−2 showed significant negative correlation with AE_N_, PEP_N_, Apparent RE_N_, and Accumulated RE_N_.

In addition, wheat yield showed significantly positive correlation with Nitrospirota and Elusimicrobiota, but was negatively associated with Armatimonadota and Methylomirabilota.

## 3. Discussion

### 3.1. Effects of Long-Term Straw Incorporation and Manure on Wheat Yield and N Use Efficiency

Straw incorporation and manure application not only increased winter wheat yield, but improved nitrogen use efficiency (NUE) (Table 1). In this study, straw incorporation treatments (SNP and SNPM) increased yield by 21.15–35.14% than no straw incorporation, and manure treatments (NPM and SNPM) increased yield by 1.52–13.25%, aligning with reports that straw and manure improved grain yields [12,13,51]. SNPM treatment had the maximum yield, aligning with findings that manure in combination with straw incorporation could improve productivity [30,58]. This could be due to the fact that straw and manure released more nutrients, and improved soil fertility and yields. Additionally, compared with NP, the nitrogen use efficiency indicators showed that AE_N_ significantly increased from 36.83% to 103.41%, PEP_N_ from 13.25% to 37.20%, RE_N_ from 17.66% to 92.24%, and accumulated RE_N_ from 8.86% to 35.16% in the NPM, SNP, and SNPM treatments, respectively. The higher N use efficiency of SNP and SNPM were attributed to soil fertility improvement through higher wheat straw and manure application.

### 3.2. Effects of Long-Term Straw Incorporation and Manure on Soil Properties

Long-term straw incorporation and manure significantly affected soil properties (Table 1). Notable differences in soil properties were observed among straw incorporation and manure. The OM, TP, AN, AP, and AK contents in the straw incorporation treatments (SNP and SNPM) were higher than those in the no-straw treatments (NP and NPM) (*p* < 0.05). This was aligned with the results of both Cao [12] and Cui [13]. The OM, TP, AN, AP, and AK contents in manure treatments (SNPM and NPM) were higher than those in no-manure treatments (NP and SNP) (*p* < 0.05); this confirmed the findings of Wen. [50] and Wu. [59]. Our study demonstrated that nutrients in manure treatments (NPM) were higher than in the straw treatment, because the organic matter in organic manure decomposes more easily and releases more nutrients than straw [60]. The OM, TP, AN, AP, and AK contents in the SNPM treatment were significantly higher than those in the CK treatment (*p* < 0.05). Akhtar et al. [61] reported that manure combination with straw significantly improved soil nutrient accumulation such as N, P, and K. Liang et al. [62] also reported that livestock manure in combination with straw could improve soil organic carbon content and soil quality in the southern Loess Plateau. These findings were in concordance with our results. Compared to NP, the straw incorporation in combination with manure significantly increased the OM, TP, AN, AP, and AK contents (Table 1). The reasons for this increase phenomenon were related to the fact that manure and straw could provide abundant organic matter to the soil and improve soil structure [63,64]. Long-term chemical fertilizer and manure decreased the soil pH from 8.07 in the CK treatment to 7.86 in the SNPM treatment, which may be attributed to the release of H^+^ or organic acids by N forms transformed or organic fertilizer decomposed [26,65,66].

### 3.3. Effects of Long-Term Straw Incorporation and Manure on Soil Bacterial Diversity

Many studies have confirmed that soil microbial diversity is altered by different fertilizer types [52,60,67] and that manure has a marked effect on microbial diversity than chemical fertilizers [68,69]. In this study, compared with the CK, the bacterial alpha-diversity increased by different degrees in the long-term fertilization, which was verified by previous research [70], but, compared with chemical fertilizer (NP), straw incorporation and manure decreased bacterial diversity, aligning with the findings of Tian et al. [57]. Compared with mineral fertilization, soil microbial diversity may vary with organic amendments [71,72]. This may be attributed to the fact that soil microbial diversity is affected by microbial groups, organic amendment types, experimental conditions, climate, initial soil properties, and crop type [52,53,73,74].

### 3.4. Effects of Long-Term Straw Incorporation and Manure on Soil Bacterial Communities

Long-term fertilization regimes can alter soil properties and lead to variations in soil microbial communities; these microbial changes, in turn, can also influence soil properties, because microbial communities play a key role in nutrient cycling [75]. Microbial taxa defined at the phylum level can show the ecological consistency of the microbial groups [74]. *Proteobacteria*, *Acidobacteriota*, *Actinobacteriota*, *Chloroflex*, *Bacteroidota*, *Planctomycetota*, *Gemmatimonadota*, *Armatimonadota*, *Firmicutes*, *Methylomirabilota*, and *Myxococcota* were the predominant bacterial phyla in this study, accounting for 92.76–93.66%. Of these, *Proteobacteria*, *Acidobacteria*, *Actinobacteria*, and *Chloroflexi* were the most abundant bacterial phyla, accounting for 65.8–69.1%, which was consistent with previous reports on agricultural soil [74,76,77]. As the most abundant bacterial community, *Proteobacteria* plays an important role in soil P and K nutrient cycling [69,78]. Compared to CK, the relative abundance of *Proteobacteria* increased by 15.34%, 9.56%, 7.08%, and 15.91% in the NP, NPM, SNP, and SNPM treatments, respectively. *Acidobacteria* belongs to the oligotrophic bacteria and thrives in resource-poor soils [79,80]. In this study, the relative abundance of *Acidobacteria* significantly decreased by 9.02–22.49%% in the NP, NPM, SNP, and SNPM treatments, compared with CK. *Actinobacteria* can mineralize and regulate the soil P cycle [81], and secrete different antibiotics to gradate recalcitrant compounds and suppress pathogenic microorganisms [82]. The relative abundance of *Actinobacteria* phylum significantly increased by 27.25–48.69% in NP, NPM, SNP, and SNPM, compared with CK. In contrast, straw incorporation and manure significantly decreased *Chlorofexi* relative abundance by 7.37–23.03% and the size of soil organic matter negatively correlated with the size of *Chlorofexi* (Figure 7), which is probably related to the fact that most *Chlorofexi* are oligotrophic populations and grow in nutrient-poor conditions [83]. Thus, soil nutrients increasing through straw incorporation treatment promote copiotrophic bacterial growth and restrict the growth of oligotrophic bacteria [80,84]. *Bacteroidetes* can decompose organic matter and improve soil fertility [85]. *Bacteroidetes* was significantly higher in CK, while *Bacteroidetes* decreased by 10.39%, 8.47%, and 7.65% in the NP, NPM, and SNPM treatments, compared with CK, possibly because of lower organic matter content. *Firmicutes* can restrict soil pathogen growth and are abundant in healthier soil environments [6]. Our results showed that NPM, SNP, and SNPM significantly improved the *Firmicutes* relative abundance, indicating that the manure and straw-treated soil was healthier.

Different treatments have unique bacterial communities. LEfSe analysis revealed that 95 significant biomarkers were detected in the bacterial microbial community in this study. *Proteobacteria* and *Acidobacteriota* were significantly enriched in CK. *Actinobacteriota* and *Chloroflexi* were significantly enriched in NP. *Entotheonellaeota* and *Proteobacteria* were significantly enriched in SNP. *Proteobacteria*, *Firmicutes*, and *Chloroflexi* were significantly enriched in NPM. *Proteobacteria* and *Acidobacteriota* were significantly enriched in SNPM.

The organic manure combination with chemical fertilizers obviously improved nutrient content and altered microbial structure in the rhizospheric soil [28,59]. Compared with chemical fertilizer alone, organic manure and straw significantly changed the soil microbial community structure [51,86]. Our cluster and PCoA analyses showed that soil bacterial communities had distinct similarities in different fertilization treatments; manure-treated (NPM and SNPM) treatments clustered together, and clearly differed from the no-manure-treated (NP and SNP) treatments and the CK treatment. Rhizospheric microorganisms utilized organic manure more readily than straw, resulting in higher bacterial biomass in NPM and SNPM compared to SNP. This is because organic fertilizer provides richer nutrients for microbial activities and essential elements for different microbial species than straw [87,88].

### 3.5. Bacterial Community Relationships with Soil Properties, Yield, and NUE

The soil substrate and nutrients usually regulate soil microbial functions [89]. Straw incorporation and manure could alter soil physicochemical properties such that these changes could influence microbial dominance phyla abundance directly or indirectly in turn, among which soil pH is a generally important factor (Figure 7). The redundancy analysis (RDA) results revealed that the bacterial community structure was obviously correlated with pH, TP, AP, OM, AK, and AN. This was consistent with the report by Shu [52] that soil pH change dominated microbial community structure variations by organic amendments. Microbial community structure may play a key role in regulating crop yield, and had significant positive relationships with microbial functionality and crop yield [52]. Application of continuous manure enhances the relationships between soil microbial function and crop yield [51]. Pearson analysis revealed that *Abditibacteriota* was significantly negatively correlated with soil TN. *Chloroflexi* was significantly negatively correlated with OM, TN, TP, AN, AP, AK, and grain yield. The phyla *Nitrospirota* is nitrite oxidizing metabolisms [90]. *Nitrospirota* was significantly positively correlated with OM, TP, AP, and wheat yield. Wheat yield was significantly positively correlated with *Elusimicrobiota*, and negatively associated with *Acidobacteriota*, *Armatimonadota*, *Cyanobacteria*, and *Methylomirabilota*. The interpretation was that organic amendments may lead to specific functional species with higher relative abundance and diversity to enhance plant defense, promote nutrient absorption, and ultimately increase crop yields [91].

## 4. Materials and Methods

The long-term winter wheat–summer maize field experiment was initiated in 2007 at the Niujiawa Agricultural Experimental station, Cotton Research Institute, Shanxi Agricultural University, located in Xia County, Yuncheng city, Shanxi province, China (35°110′ N, 111°050′ E). The site information has been described in a previous study by Li [40]. The climate at the site is characterized as a temperate monsoon, with an average annual temperature of 13.3 °C and average annual precipitation of 525 mm. The soil type is cinnamon soil with a silty clay loam texture (17.5% clay, 28.0% sandy soil, and 54.5% silty sand). The initial basic topsoil layer (0–20 cm) properties were as follows: pH 8.15, organic matter (OM) 10.6 g/kg, total nitrogen (TN) 0.89 g/kg, total phosphorus (TP)1.08 g/kg, available nitrogen (AN) 56.9 mg/kg, available phosphorus (AP) 13.1 mg/kg, and available potassium (AK) 159.6 mg/kg.

The field experiment used a completely randomized design with three replications. The size of each plot was 60 m^2^. The five treatments included in this study were (1) CK, no fertilization control; (2) NP, inorganic N and P fertilizers; (3) NPM, mineral N and P fertilizers plus chicken manure; (4) SNP, mineral N and P fertilizers plus maize straw; and (5) SNPM, mineral N and P fertilizers plus maize straw and chicken manure. Mineral N and P fertilizers and manure were applied as urea, calcium super-phosphate, and chicken manure with total 450 kg N/(ha·yr), 148.5 kg P/(ha·yr), and 9 t/(ha·yr) (dry weight) to two crops, respectively. Chicken manure and P fertilizer were added before wheat sowing, two-thirds the of urea were applied as a basal dose before sowing, the one-third of the urea was top dressed at the jointing stage of each crop. After maize was harvested, maize straw was chopped into 10 cm-length pieces, and incorporated into the soil prior to wheat sowing for the straw incorporation treatments (SNP and SNPM). The amount of straw incorporation was 10.5 t/ha each year. The wheat straw of all treatments was incorporated into the soils after being chopped. A rotary tillage operation till was performed by machine before sowing and to mix the mineral fertilizers, straw, and manure into the soil at a depth of about 10–15 cm.

### 4.1. Sampling Collection and Chemical Analysis

Grain yield was determined by harvesting three 6 m^2^ sections from the center of each plot. Ten soil samples were collected from each plot (0–20 cm depth layer) after the wheat harvest in June 2021 using an auger and mixed to form a homogeneous sample. After mixing, the sample was divided into two parts. A portion of the soil samples was stored at −80 °C immediately, and the other soil samples were air-dried for soil property analyses.

Wheat samples were divided into grain and straw and subsequently dried at 70 °C. Total nitrogen (TN) was analyzed by the Kjeldahl digestion method using a SKD-800 automatic analyzer (Shanghai Peiou Analytical Instrument Co., Ltd., Shanghai, China). Soil organic matter (OM) was determined using a spectrophotometer method after oxidation with K_2_Cr_2_O_7_ (Shanghai Metash Instruments Co., Ltd., Shanghai, China). Soil available nitrogen (AN) was determined using the hydrolysis–alkaline diffusion method. Total phosphorus (TP) was quantified using the HClO_4_-H_2_SO_4_ method, and available phosphorus (AP) was determined using molybdenum blue colorimetry after 0.5 mol/L NaHCO_3_ extraction. Available potassium (AK) was determined by the flame photometric method after ammonium acetate extraction using an AP1302 flame spectrophotometer (Shanghai Instruments Group Co., Ltd., Shanghai, China). Soil pH was measured using a 1:2.5 soil deionized water suspension by a FE20 pH meter (Mettler Toledo, Shanghai, China).

The N use efficiency (NUE) was calculated with four indexes, as follows [92,93]:

Apparent recovery efficiency of N (Apparent RE_N_, %) = (N_uptake_−0 N_uptake_)/Input N × 100%

Accumulated recovery efficiency of N (Accumulated RE_N_) = (accumulated N_uptake_−accumulated 0 N_uptake_)/accumulated N_uptake_ × 100%

Agronomic efficiency of N (AE_N_, kg/kg) = (N_yield_ − 0 N_yield_)/Input N

Partial factor productivity of N (PFP_N_, kg/kg) = N_yield_/Input N

In the equations, N_uptake_ and 0 N_uptake_ indicate N uptake from N, 0 treatment plots, respectively, N_yield_ represents grain yield obtained in the N application treatments, and 0 N_yield_ represents grain yield obtained in the control plots. The Input N is the total amount of N applied in treatments during wheat growth.

### 4.2. DNA Extraction and Illumina MiSeqsequencing

DNA was extracted from a 0.5 g freeze-dried soil sample using the E.Z.N.A.^®^ Soil DNA Kit (Omega Bio-tek, Norcross, GA, USA) according to the E.Z.N.A.^®^ Soil DNA Kit (Omega Bio-tek, Norcross, GA, USA). The quality of the extracted DNA was determined with a NanoDrop 2000 spectrophotometer (Thermo Scientifc, Waltham, USA). The primers 515F 5′-barcode- GTGCCAGCMGCCGCGG)-3′ and 907R 5′-CCGTCAATTCMTTTRAGTTT-3′ were used to amplify the V4-V5 region of the bacteria 16S rRNA gene.

MiSeqsequencing of purified amplicons was performed on an Illumina MiSeq platform (Illumina, San Diego, CA, USA) at Shanghai Biozeron Technology Co., Ltd., Shanghai, China. The sequencing data was analyzed using the Quantitative Insights into Microbial Ecology (QIIME) pipeline (version 1.17). The operational taxonomic units (OTUs) were clustered with 97% similarity levels using UPARSE (version 7.1 http://drive5.com/uparse/ (accessed on 9 October 2021)) and chimeric sequences were identified and removed using UCHIME. The taxonomy of each 16S rRNA gene sequence was analyzed using the RDP Classifier algorithm (http://rdp.cme.msu.edu/ (accessed on 9 October 2021)) against the Silva (SSU123) 16S rRNA database using a confidence threshold of 70%.

### 4.3. Statistical Analysis

The Chao1 index, Shannon index, ACE index, and evenness index of the bacteria were calculated using Mothur (version v.1.30.1). Based on the Bray–Curtis distance matrix, principal coordinates analysis (PCoA) and hierarchical cluster analysis were performed to reveal the differences in the bacterial community between treatments. The R software (v 4.0.3) package “vegan” was used for redundancy analysis (RDA) to evaluate the relationships between the bacterial community and soil properties. LEfSe used linear discriminant analysis (LDA) to identify differences in the bacterial communities between treatments. The generated data were analyzed on the free online platform of Shanghai Biozeron Technology Co., Ltd., Shanghai, China.

One-way analysis of variance (ANOVA) and the t-test were conducted using IBM SPSS Statistics (v.27.0) software (IBM Corp, Armonk, NY, USA). The LSD method was used to determine the significance level with an α of 0.05. Pearson correlation analysis of bacterial community with soil properties, yield, and NUE indicators was performed using Origin (v.2021) (Origin Lab Corp, Northampton, MA, USA) and Adobe Illustrator (v.2021).

## 5. Conclusions

In this study, compared with CK, long-term straw incorporation and manure application alone or in combination (NPM, SNP, and SNPM) significantly improved chemical properties, wheat yields, and N use efficiency and optimized soil bacterial community composition and alpha diversities; in particular, the SNPM treatment was the best. More importantly, the SNPM treatment enhanced soil fertility, thereby optimizing wheat yields, N use efficiency, and bacterial community composition through improving chemical properties. Therefore, straw incorporation in combination with manure treatment deserves to be applied in more areas as a sustainable farming strategy.

## Figures and Tables

**Table 1:** Wheat grain yield and NUE indicators in the different treatments.

Treatments	Grain Yield (kg/ha)	AE_N_ (kg/kg)	PEP_N_ (kg/kg)	Apparent RE_N_ (%)	Accumulated RE_N_ (%)
NP	5402.7 ± 353.17 c	8.64 ± 1.98 c	24.01 ± 1.57 c	33.80 ± 5.87 b	45.59 ± 5.09 c
NPM	6118.61 ± 224.71 b	11.82 ± 0.8 b	27.19 ± 1.00 b	39.77 ± 2.36 b	49.83 ± 1.76 b
SNP	7301.40 ± 575.01 a	17.07 ± 2.88 a	32.45 ± 2.56 a	64.98 ± 9.08 a	61.70 ± 3.84 a
SNPM	7412.59 ± 512.56 a	17.57 ± 2.6 a	32.94 ± 2.28 a	59.62 ± 7.69 a	59.68 ± 3.69 a
CK	3459.00 ± 144.90 d	-	-	-	-

Note: Different lowercase letters within a column indicate significant differences at 0.05 level.

**Table 2:** Effects of straw incorporation and manure treatments on soil chemical properties.

Treatments	pH	OM g/kg	TN g/kg	TP g/kg	AN mg/kg	AP mg/kg	AK mg/kg
NP	8.05 ± 0.02 ab	9.50 ± 0.50 c	13.10 ± 6.50 a	4.03 ± 0.56 c	60.67 ± 10.69 c	68.79 ± 4.89 c	187.99 ± 5.63 b
NPM	7.98 ± 0.04 b	11.50 ± 0.10 b	17.70 ± 4.30 a	6.33 ± 0.36 b	102.33 ± 10.69 b	270.42 ± 6.24 b	260.88 ± 34.24 c
SNP	7.99 ± 0.02 b	11.20 ± 0.30 b	18.70 ± 5.80 a	3.35 ± 0.17 cd	102.50 ± 14.57 b	55.71 ± 3.85 c	220.82 ± 12.65 c
SNPM	7.86 ± 0.03 c	16.50 ± 1.20 a	18.70 ± 1.60 a	15.54 ± 0.72 a	165.67 ± 14.57 a	345.30 ± 22.50 a	447.30 ± 29.13 a
CK	8.07 ± 0.08 a	9.08 ± 0.40 c	12.10 ± 5.80 a	2.66 ± 0.15 d	67.33 ± 14.57 c	24.02 ± 2.37 d	140.89 ± 8.22 d

Note: Different lowercase letters in a column indicate significant differences at 0.05 level.

**Table 3:** Richness and diversity indices of soil bacteria under fertilization regimes.

Treatments	Community Richness Index	Chao1 Index	Shannon Index	ACE Index
NP	4320.33 ± 8.50 a	4993.09 ± 73.30 a	10.45 ± 0.03 a	4851.89 ± 381.37 a
NPM	4152.33 ± 209.09 ab	4773.96 ± 147.46 ab	10.27 ± 0.05 ab	4869.02 ± 161.74 a
SNP	3866.33 ± 266.27 bc	4784.61 ± 5.42 ab	10.22 ± 0.25 ab	4696.24 ± 183.87 a
SNPM	3989.00 ± 128.95 abc	4659.32 ± 151.57 b	10.14 ± 0.10 ab	4735.98 ± 163.06 a
CK	3619.00 ± 303.85 c	4304.08 ± 252.27 c	10.01 ± 0.21 b	4365.85 ± 195.00 a

Note: Different lowercase letters in a column indicate significant differences at 0.05 level.

## Data Availability

Data are contained within the article.
